# Supplementary material for: Rift Valley fever outbreak among animal handlers in Kinyogoga Village, Nakaseke District, Central Uganda: A case study
Source: PLOS Glob Public Health. 2025 Aug 25;5(8):e0003420. doi: 10.1371/journal.pgph.0003420 (PMC12377559; doi:10.1371/journal.pgph.0003420)
Supplement: Attachment — Submitted filename: Response to Reviewers.docx [file pgph.0003420.s004.docx]

**TELEPHONE**

**General office: 340874/231563/9**

**PS’s office: 340872**

**TELEFAX: 231584**

**TELEX: 61372 HEALTH UGA.**

**In ANY CORRESPONDENCE ON THIS SUBJECT PLEASE QUOTE NO. ADM. 110/269/024**

**MINISTRY OF HEALTH**

**Plot 6 Lourdel Rd, Wandegeya**

**P.O. Box 7272 KAMPALA, UGANDA**

**7^th^ October 2024**

Academic Editor
PLOS Global Public Health

**Re: Resubmission of the edited manuscript** PGPH-D-24-01296

Dear Dr Chowdhury Sukanta,

I would like to extend my gratitude to you, the editorial team, and the reviewers for reviewing our manuscript entitled " Rift Valley fever outbreak among animal handlers linked to contact with infected animal products in Kinyogoga Village, Nakaseke District, Central Uganda, June–July 2023."

We have addressed all the comments and suggestions, which have been highlighted in red in the revised manuscript. Additionally, we have provided a point-by-point response to all the reviewers' comments, attached to this letter.

The feedback was invaluable and has significantly improved the quality of our manuscript. We hope you find it worthy of publication in the Journal of PLOS Global Public Health.

We look forward to your response at your earliest convenience.

Yours sincerely,

……………………….

Mariam Komugisha

Fellow, Uganda National Institute of Public Health

**Point-by-Point Response to Editor and Reviewer Comments**

PGPH-D-24-01296 (PLOS Global Public Health)

Rift Valley fever outbreak among animal handlers linked to contact with infected animal products in Kinyogoga Village, Nakaseke District, Central Uganda, June–July 2023

**Editor’s comments:**

Journal Requirements:

1. Your funding information on the submission form indicates, Grant Recipient Dr Alex Riolexus Ario. Please indicate by return email the full and correct funding information for your study and confirm the order in which funding contributions should appear. Please be sure to indicate whether the funders played any role in the study design, data collection and analysis, decision to publish, or preparation of the manuscript.

**Response:** The email has been sent

2. Please provide separate figure files in .tif or .eps format.

For more information about figure files please see our guidelines:  LINK

<https://journals.plos.org/globalpublichealth/s/figures> 
<https://journals.plos.org/globalpublichealth/s/figures#loc-file-requirements>

**Response:** Sent separate figure files in .tif format

3. In the online submission form, you indicated that "the data sets can be made available upon reasonable request from the corresponding author".

All PLOS journals now require all data underlying the findings described in their manuscript to be freely available to other researchers, either 
1. In a public repository, 
2. Within the manuscript itself, or 
3. Uploaded as supplementary information.

This policy applies to all data except where public deposition would breach compliance with the protocol approved by your research ethics board. If your data cannot be made publicly available for ethical or legal reasons (e.g., public availability would compromise patient privacy), please explain your reasons by return email and your exemption request will be escalated to the editor for approval. Your exemption request will be handled independently and will not hold up the peer review process, but will need to be resolved should your manuscript be accepted for publication. One of the Editorial team will then be in touch if there are any issues.

**Response:** This has been noted. The data set has been uploaded as supplementary information

4. Figure 1 and 2: please (a) provide a direct link to the base layer of the map (i.e., the country or region border shape) and ensure this is also included in the figure legend; and (b) provide a link to the terms of use / license information for the base layer image or shapefile. We cannot publish proprietary or copyrighted maps (e.g. Google Maps, Mapquest) and the terms of use for your map base layer must be compatible with our CC-BY 4.0 license.

Note: if you created the map in a software program like R or ArcGIS, please locate and indicate the source of the basemap shapefile onto which data has been plotted.

If your map was obtained from a copyrighted source please amend the figure so that the base map used is from an openly available source. Alternatively, please provide explicit written permission from the copyright holder granting you the right to publish the material under our CC-BY 4.0 license.

Please note that the following CC BY licenses are compatible with PLOS license: CC BY 4.0, CC BY 2.0 and CC BY 3.0, meanwhile such licenses as CC BY-ND 3.0 and others are not compatible due to additional restrictions.

If you are unsure whether you can use a map or not, please do reach out and we will be able to help you. The following websites are good examples of where you can source open access or public domain maps: 
* U.S. Geological Survey (USGS) - All maps are in the public domain. ([http://www.usgs.gov](http://www.usgs.gov/)) 
* PlaniGlobe - All maps are published under a Creative Commons license so please cite “PlaniGlobe, [http://www.planiglobe.com](http://www.planiglobe.com/), CC BY 2.0” in the image credit after the caption. (<http://www.planiglobe.com/?lang=enl>) 
* Natural Earth - All maps are public domain. (<http://www.naturalearthdata.com/about/terms-of-use/>)

**Response:** Addressed as guided by the editor

**Reviewers' comments:**

Reviewer-1

Authors Komugisha et al. describe a very specific RVFV outbreak in Uganda with human cases. Description of the clinical impacts of this outbreak focus on only two of the few cases (N = 8), as these were the ones they could confirm via RT-PCR. No other diagnostic testing to confirm exposure (such as serological testing) was utilized, therefore the true scope of this outbreak is not described by this paper. Similarly, animal testing is not described. While this paper is well written, it suffers from redundancy and does not investigate beyond the surface. I would not describe this as a research article, and may be better presented as a case study.

**Response:** Absolutely. Our work presents a case study of an RVF outbreak in Nakaseke, with the diagnosis primarily focused on the case patients. We have revised both the title and the content to better reflect this perspective. Thank you for highlighting this.

Specific comments and edits:
1. This paper is light on references, especially in the introduction.

**Response:** We have added references as suggested

2. Page 3, line 62: should read "the infection" rather than "the disease has an incubation period". The disease is symptomatic presentation resulting from infection. Rather, the virus has an incubation period before the symptoms commence.

**Response:** We have revised the sentence as suggested

3. Page 3, lines 67 and 68: IgM is not defined, but Immunoglobulin G is fully spelled out without abbreviation. Review for consistency.

**Response:** We have revised the sentence as suggested

4. Page 4, lines 74 and 80: Rift Valley Fever Virus has already been defined previously, and should therefore only be referred to as RVFV following initial mention.

**Response:** We have revised the sentence as suggested

5. Page 4, paragraph starting on line 74: No mention of vaccines for livestocks is included, and this feels like a major oversight. If the vaccines are not used or not available to farms and herds in this region, this should be discussed. In fact, there is no mention of animal vaccination at all in the entire paper!

**Response:** As suggested, we have, we have improved this section by discussing vaccines use in livestock

6. Figure 1: check map for text clarity, it prints pixelated for me in it's submitted size/resolution.

**Response:** As suggested, we drew another map with clear texts

7. Page 6, section on descriptive epidemiology is lacking detail, so it is impossible to assess **the actual work done beyond basic arithmetic to calculate percentages. No statistics.**

**Response:** The reviewer is correct that we relied on basic arithmetic methods. This limitation was due to the restricted data available, as this is a case study. We have acknowledged this as part of the study’s limitations.

8. Pages 6 and 7, ethical considerations section causes concern. No published consent forms or questions, no discussion of avoiding coercion while obtaining strictly verbal consent. What language was used? No discussion of who was not included, or if any individuals were approached and declined. Just says "all respondents were above the age of 18 and gave verbal consent for interviews." Using COVID as a reason for only obtaining verbal consent in 2023 is not convincing enough. Consenting practices are important.

**Response:** As suggested, we revised the ethical consideration section, we included the detail in this section such as some of the information in the consent form, language used, individuals included.

9. Page 7, line 157: were participants asked about a single instance of fever or recurrent fevers? What about temperature of fevers? RVF typically is associated with high fever.

**Response:** Patients were asked about a single instance of high fever

10. Page 7, line 160-161: "The time to death from symptom onset was 16 days." This section is confusing as currently written. Is this referring to the individual human that died, or the livestock mentioned in the previous sentence? Revise this section for clarity.

**Response:** The reference to "16 days from onset of symptoms to death" pertains to the human case, not the animals. We have revised this section to clarify.

11. Figure 3: what are the green panels in this figure? Not described anywhere.

**Response:** The green panel in Fig 3 was a forest reserve. We decided to delete it from the map

12. Page 9, lines 208-212: How many individuals had a differential diagnosis for brucellosis and typhoid? Both confirmed cases were initially misdiagnosed for these two infections, plus one mention of gastritis or gastric ulcer. Is this an issue with the clinical teams not being knowledgeable about RVFV, or not performing diagnostic testing? Further description is warranted in the discussion, because out of the two confirmed cases of this outbreak study, one person died, and the way it was described, it seems it was from medical negligence.

**Response:** As suggested by the reviewer, we included further description in the discussion with the focus on the two confirmed cases who were initially misdiagnosed for brucellosis and typhoid. Three case-patients were initially misdiagnosed for the two infections.

13. Page 10, line 216: I'm not sure it is valuable to compare basic percentages with such a small number of surveyed individuals and no further testing performed. The extremely small N is likely the contributing factor to the higher fatality rate compared to Sudan and all other outbreaks that weren't mentioned.

**Response:** Thank you for pointing this out. We decide decided to delete this statement in the revised version. We included this in the study limitation

14. Page 10, line 219: mention of CCHF, was there any investigation to whether co-infection was possible here? Any reported co-infections that suggest this is a possibility? Concerns about serological diagnoses? This sentence seems like an afterthought.

**Response:** Thank you for pointing this out. It is true the coinfection with CCHF was an oversight due to previous reports of the disease in the area. In the revised manuscript, we have deleted this statement and improved the clarity of the statement.

15. Page 10, lines 221-225: again, no mention of vaccines for livestock, also failed to mention mosquito abatement.

**Response:** Thank you for pointing this out. In the revised section we mention vaccine use on the affected farms, and the need for vaccination of animals and mosquito control.

16. Page 10, lines 238 - 240: mention of herdsmen having increased exposure is complicated because there are much more thorough longitudinal studies that show distinct roles in Kenya (Cook et al.), and butchery was the most risky, well above herding. This study fails to discuss the challenges distinguishing these roles in their specific population due to overlapping roles/behaviors. Would have liked to see a more thorough discussion on this and how performing multiple duties can increase risk of RVFV exposure.

**Response:** Thank you for pointing this out. Unlike traditional herding practices, herdsmen in Uganda also engage in activities such as milking, slaughtering, and assisting with animal reproduction, which increases their risk. In the revised manuscript, we have included a thorough discussion on how performing multiple high-risk activities elevates their exposure to Rift Valley Fever virus (RVFV).

17. Page 11, line 253: study limitations are an afterthought as well. This study has so many limitations that are not described. Why were individuals with no or mild symptoms excluded, when RVFV infection has a high rate of asymptomatic cases? How can the epidemiology of this outbreak be investigated if it is based on only two confirmed cases? Much of these findings seem like general assumptions.

**Response:** Thank you for pointing this out.

18. Page 11, line 261 and beyond: why is the education factor only now being mentioned in this paper? No documentation or descriptions of methods used for educating are included. Do the authors plan to follow up at a later date to track efficacy of their educational efforts?

**Response:** Thank you for pointing this out. Education was a public health action undertaken to control the outbreak in the affected area. The authors have no plan to follow up to track efficacy of the education efforts. In the revised manuscript we deleted the public health actions and added the recommendations.

Reviewer-2:

General comments
These are valuable findings and raise awareness of the importance of having the human and animal health sectors work together to tackle zoonotic disease. In addition, of increasing awareness among clinical and veterinary personal of potential circulating pathogens, having robust surveillance systems, and having appropriate diagnostics in place for the timely and accurate detection of disease to reduce/prevent morbidity and mortality in both humans and animals.
I would recommend proof-reading the manuscript again for English grammar.

**Response:** Thank you. Addressed as guided

Abstract
Line 25: RVF virus

**Response:** Revised as suggested by the reviewer

Line 26: lower case for “reverse transcriptase”

**Response:** This has been changed as revised by the reviewer

Line 27: “identify the source, estimate the magnitude, and …”

Response: Revised as suggested by the reviewer

Line 32: RVF virus

Response: Added the word virus

Introduction
Line 67-68: Immunoglobulin (Ig) M…..IgG

Response: Spelled out Immunoglobulin M on first use and summarized IgG

Line 74: Rift Valley fever virus = RVFV, check throughout manuscript

**Response:** This has been changed throughout the manuscript

Line 88: Which animals? Countrywide or a specific region?

**Response:** Specified the animals. The animals include: cattle, sheep and goats. The sero-prevalence of 11% was country-wide.

Methods
Line 117: rates

**Response:** Revised as suggested by the reviewer.

Line 123: “The proportion of case-patients were calculated for sex, occupation and symptoms”

**Response**: This sentence has been revised

Line 133: herds men or herdsmen? – check throughout manuscript

**Response:** This has been changed in the revised manuscript

Line 147: Is verbal consent sufficient in Uganda?

**Response:** Yes, verbal consent in Uganda is sufficient if the participants are illiterate. This has been revised in the revised manuscript.

Results
Line 153: Be careful about reporting CFR with a small sample size, only a single death and 8 cases. Add to limitations.

**Response:** Thank you for pointing this out. This was added to the limitations of the study

Line 162: Rift Valley fever – check correct use of capitals throughout manuscript

**Response:** This has been revised in the revised manuscript

Line 180: “…year, which started in early June and continued throughout…”

**Response:** Noted. This has been revised as suggested by the reviewer

Line 182: When/how was the case detected? Perhaps rephrase the sentence.

**Response:** This has been rephrased

Line 202: no comma after fetuses (also plural)

**Response:** Revised as suggested by the reviewer

Line 204: “…and regularly milked…”

**Response:** This has been revised

Line 206: post-mortems

Response; added “s” on post-mortem

Discussion
Line 216: Be careful about reporting CFR with a small sample size. Perhaps better to just describe the single case, that the delay in correct diagnosis may have contributed to this patients death. Add to limitations that the small sample size limits statistical analysis to identify risk factors.
**Response:** Addressed as guided as guided by the reviewer

Line 240: Aedes spp and Culex spp. (scientific names in italics

**Response:** We changed to italics

Line 260: “…weak, impeding timely detection, which…”

**Response:** We revised the sentence as suggested by the reviewer
