# Supplementary material for: Rift Valley fever outbreak among animal handlers in Kinyogoga Village, Nakaseke District, Central Uganda: A case study
Source: PLOS Glob Public Health. 2025 Aug 25;5(8):e0003420. doi: 10.1371/journal.pgph.0003420 (PMC12377559; doi:10.1371/journal.pgph.0003420)
Supplement: Attachment — Submitted filename: My comment.docx [file pgph.0003420.s003.docx]

Minor comments:

- Abstract: line 28. The sentence is not correct grammatically (A suspected case was a sudden onset of fever…….). You consider to write this way (We defined a suspected case as an individual with the sudden onset of fever (≥38°C)………….)
- The case definition is less clear to me. Did each suspected patient show all clinical signs including sudden onset of fever (≥38°C) and ≥2 of headache, skin rash, muscle/joint pain, intense fatigue, dizziness, coughing, abdominal pain, or bleeding? Or showed few clinical signs? Clarify the case definition in abstract and method section. You mentioned “bleeding”. Please specify whether it was nose bleeding/mouth bleeding/others…
- Median age for whom? Suspected cases or confirmed cases? Clarify
- All case-patients mean 8 cases or 2 cases? Clarify this in the abstract.
- hemorrhagic symptoms: where was it shown? Skin or conjunctiva?
- Diagnosed brucellosis, typhoid and gastritis through clinically or laboratory tests?
- Specify “infected animals’ products”. Meat/milk? What types of animals (livestock/wild)?
- Descriptive epidemiology section (method): why you mentioned case patients and index case separately? Case patients included index case. You can mention index case and secondary cases
- Why you included animal sickness history under environmental section? I have not seen any environmental variables. Revise this both method and result section.
- Add detail information about RT-PCR TaqMan assay
- Result: The fatality was the index case-patient (animal husbandry officer) who developed….Revise the sentence.
- Limitation: According to your study result, the CFR was 50%. Clarify it.
- Conclusion: The suspicion index for VHFs is low among clinicians…..this is not clear to me. Who are clinicians? Medical doctor or vet. Doctor?
- Animal disease surveillance system is weak…This is vague statement. Is there ongoing surveillance? Or inadequate surveillance. Revise this section.
- Training for clinician… why clinician? How about veterinarian/animal handler/quack?
