# Supplementary material for: Rift Valley fever outbreak among animal handlers in Kinyogoga Village, Nakaseke District, Central Uganda: A case study
Source: PLOS Glob Public Health. 2025 Aug 25;5(8):e0003420. doi: 10.1371/journal.pgph.0003420 (PMC12377559; doi:10.1371/journal.pgph.0003420)
Supplement: Attachment — Submitted filename: Response to Reviewers1.docx [file pgph.0003420.s005.docx]

**TELEPHONE**

**General office: 340874/231563/9**

**PS’s office: 340872**

**TELEFAX: 231584**

**TELEX: 61372 HEALTH UGA.**

**In ANY CORRESPONDENCE ON THIS SUBJECT PLEASE QUOTE NO. ADM. 110/269/024**

**MINISTRY OF HEALTH**

**Plot 6 Lourdel Rd, Wandegeya**

**P.O. Box 7272 KAMPALA, UGANDA**

**24^th^ June 2025**

Academic Editor
PLOS Global Public Health

**Re: Resubmission of the edited manuscript** PGPH-D-24-01296

Dear Dr Chowdhury Sukanta,

I would like to extend my gratitude to you, the editorial team, and the reviewers for reviewing our manuscript entitled " Rift Valley fever outbreak among animal handlers in Kinyogoga Village, Nakaseke District, Central Uganda: a case study."

We have addressed all the comments and suggestions, which have been highlighted in red and blue in the revised manuscript. Additionally, we have provided a point-by-point response to all the reviewers' comments, attached to this letter.

The feedback was invaluable and has significantly improved the quality of our manuscript. We hope you find it worthy of publication in the Journal of PLOS Global Public Health.

We look forward to your response at your earliest convenience.

Yours sincerely,

……………………….

Mariam Komugisa

Fellow, Uganda National Institute of Public Health

**Point-by-Point Response to Editor and Reviewer Comments**

PGPH-D-24-01296 (PLOS Global Public Health)

Rift Valley fever outbreak among animal handlers in Kinyogoga Village, Nakaseke District, Central Uganda: a case study

**Reviewers' comments:**

Reviewer-1

Minor comments:

- Abstract: line 28. The sentence is not correct grammatically (A suspected case was a sudden onset of fever…….). You consider to write this way (We defined a suspected case as an individual with the sudden onset of fever (≥38°C)………….)

**Response:** This has been revised

- The case definition is less clear to me. Did each suspected patient show all clinical signs including sudden onset of fever (≥38°C) and ≥2 of headache, skin rash, muscle/joint pain, intense fatigue, dizziness, coughing, abdominal pain, or bleeding? Or showed few clinical signs? Clarify the case definition in abstract and method section. You mentioned “bleeding”. Please specify whether it was nose bleeding/mouth bleeding/others…

**Response:** The suspected cases did not show all the signs. All suspected cases had fever with at least two of the following signs, including headache, skin rash, muscle/joint pain, intense fatigue, dizziness, coughing, abdominal pain, or bleeding. We have revised the bleeding to unexplained bleeding from any site.

- Median age for whom? Suspected cases or confirmed cases? Clarify

**Response:** The median age for both the suspected and confirmed cases. This has been revised

- All case-patients mean 8 cases or 2 cases? Clarify this in the abstract.

**Response:** This has been revised.

- hemorrhagic symptoms: where was it shown? Skin or conjunctiva?

**Response:** This has been revised

- Diagnosed brucellosis, typhoid and gastritis through clinically or laboratory tests?

**Response:** This was a clinical diagnosis. This has been revised.

- Specify “infected animals’ products”. Meat/milk? What types of animals (livestock/wild)?

**Response:** This has been revised.

- Descriptive epidemiology section (method): why you mentioned case patients and index case separately? Case patients included index case. You can mention index case and secondary cases

**Response:** This has been revised.

- Why you included animal sickness history under environmental section? I have not seen any environmental variables. Revise this both method and result section.

**Response:** This has been revised.

- Add detail information about RT-PCR TaqMan assay

**Response:** We included the detailed information about RT-PCR test performed.

- Result: The fatality was the index case-patient (animal husbandry officer) who developed….Revise the sentence.

**Response:** We have revised the sentence.

- Limitation: According to your study result, the CFR was 50%. Clarify it.

**Response:** In this study we did not report the CFR due to fewer number of case-patients. This was a limitation of this study.

- Conclusion: The suspicion index for VHFs is low among clinicians…..this is not clear to me. Who are clinicians? Medical doctor or vet. Doctor?

Response: We were referring to healthcare workers. This has been revised

- Animal disease surveillance system is weak…This is vague statement. Is there ongoing surveillance? Or inadequate surveillance. Revise this section.

**Response:** This has been revised. The surveillance system is inadequate.

- Training for clinician… why clinician? How about veterinarian/animal handler/quack?

**Response:** This has been revised.

Reviewers' comments:

Reviewer #1: The authors have done a fantastic job addressing all reviewer comments. This manuscript is much more clear after the revision, and highlights the important work conducted by the team of authors.
One small edit: Page 6, Line 128: Add a period at the end of the final sentence in this paragraph.

**Response:** We added the period.

Reviewer 2

 The authors have addressed my comments and the readability of the manuscript has greatly improved. My remaining concern is regarding the verbal informed consent. Generally, a witnessed mark or thumb-print is standard practice for consenting illiterate participants. Was verbal informed consent approved by an ethics committee?

**Response:** We obtained administrative clearance from the Ministry of Health

Additional minor comments:
Check consistency of using "RVF virus" vs "RVFV"

**Response:** This has been revised as suggested.

Line 196: should be lambs not lamb

**Response:** This has been revised.
